# Assessment and Management of Cardiotoxicity in Hematologic Malignancies

**DOI:** 10.1155/2021/6616265

**Published:** 2021-02-03

**Authors:** Anca Bojan, Tunde Torok-Vistai, Andrada Parvu

**Affiliations:** ^1^Ion Chiricuta Oncology Institute, Cluj-Napoca, Romania; ^2^Iuliu Hatieganu University of Medicine and Pharmacy, Cluj-Napoca, Romania

## Abstract

With the increasing overall survival of cancer patients due to recent discoveries in oncology, the incidence of side effects is also rising, and along with secondary malignancies, cardiotoxicity is one of the most concerning side effects, affecting the quality of life of cancer survivors. There are two types of cardiotoxicity associated with chemotherapy; the first one is acute, life-threatening but, fortunately, in most of the cases, reversible; and the second one is with late onset and mostly irreversible. The most studied drugs associated with cardiotoxicity are anthracyclines, but many new agents have demonstrated unexpected cardiotoxic effect, including those currently used in multiple myeloma treatment (proteasome inhibitors and immunomodulatory agents), tyrosine kinase inhibitors used in the treatment of chronic myeloid leukemia and some forms of acute leukemia, and immune checkpoint inhibitors recently introduced in treatment of refractory lymphoma patients. To prevent irreversible myocardial damage, early recognition of cardiac toxicity is mandatory. Traditional methods like echocardiography and magnetic resonance imaging are capable of detecting structural and functional changings, but unable to detect early myocardial damage; therefore, more sensible biomarkers like troponins and natriuretic peptides have to be introduced into the current practice. Baseline assessment of patients allows the identification of those with high risk for cardiotoxicity, while monitoring during and after treatment is important for early detection of cardiotoxicity and prompt intervention.

## 1. Introduction

Due to the advancement in cancer treatment in the last years, the overall survival of cancer patients increased significantly. Unfortunately, this has also led to increased exposure to side effects of different treatment modalities. One of the most important side effects with a major impact on survival is cardiac toxicity. Therefore, a multidisciplinary approach of these patients is necessary, in order to find a balance between the response to treatment and cardiovascular morbidity and mortality. Development of protocols for prevention and early treatment of cardiotoxicity can avoid chemotherapy withdrawal and optimize outcomes [1]. In this article, the authors aimed to review the most important cardiotoxic therapies used in hematologic malignancies, describe their mechanism of action, and summarise the imagistic and laboratory methods used for monitoring cardiotoxicity, highlighting the importance of early detection and intervention.

## 2. Therapies with Cardiotoxic Potential

Drugs with cardiotoxic potential have been classified into two groups: type I agents, which cause a dose-dependent and mainly irreversible cardiotoxicity (e.g., anthracyclines), and type II agents, whose cardiotoxicity is not dose dependent and mainly reversible (e.g., tyrosine kinase inhibitors, immunomodulatory drugs, and proteasome inhibitors) [2].

## 3. Mechanism of Cardiotoxicity

### 3.1. Cardiotoxicity of Anthracyclines

Anthracyclines are antibiotic antineoplastic agents discovered in 1963 and well known for their cardiotoxic effect. Doxorubicin and daunorubicin are two members of this class. Anthracycline-induced cardiotoxicity can have two forms:

- Early or acute, which may manifest as arrhythmia, myocarditis, pericarditis, or acute left ventricular failure; these complications resolve after withdrawal of treatment. This type of anthracycline-induced cardiotoxicity is more common in the elderly, probably due to underlying heart disease, and also in patients with large single doses of doxorubicin
- Late or chronic cardiomyopathy, with late onset of arrhythmia and ventricular dysfunction; this type of cardiotoxicity is related to the cumulative dose of doxorubicin. Studies have demonstrated that the estimated cumulative incidence of congestive heart failure was 5% at a cumulative dose of 400 mg/m^2^, 26% at a dose of 550mg/m^2^, and 48% at a dose of 700 mg/m^2^ [3, 4]

#### 3.1.1. Mechanism of Anthracycline-Induced Cardiotoxicity

Generally, cardiotoxicity is caused by myocardial cell loss, apoptosis, and necrosis, mediated by oxidative stress, but the exact mechanism of anthracycline-induced cardiotoxicity is not known. There are four proposed hypotheses:

- Iron and free radical theory, in which oxidative stress is involved due to depletion of endogenous antioxidant
- Metabolic hypothesis, in which an alcoholic anthracycline metabolite interferes with the myocardial energy pathway and intracellular calcium concentration
- Unifying hypothesis, in which an alcoholic anthracycline metabolite also causes increased calcium concentration in the myocardial fiber and damages it
- Apoptosis hypothesis, in which there is an upregulation of proapoptotic markers [5]

### 3.2. Cardiotoxicity of Cyclophosphamide

Cyclophosphamide is an alkylating agent which can cause cardiotoxicity shortly after therapy, due to a toxic effect of its metabolite on the endothelial cells. It can cause myopericarditis and myocardial necrosis and also pulmonary hypertension [5].

### 3.3. Cardiotoxicity of Tyrosine Kinase Inhibitors

Tyrosine kinase inhibitors (TKIs) have revolutionised cancer therapy, especially that of chronic myeloid leukemia. They bind to the adenosine triphosphate (ATP) binding pocket of the tyrosine kinase and transfer a phosphate group from ATP to a tyrosine residue. TKIs inhibit not only the malignant cells but also the nonmalignant cells as well, and this explains their side effects. The most common side effects are rush and diarrhea, but they also may cause cardiotoxicity. Cardiotoxicity of TKIs ranges from asymptomatic QT prolongation to decreased LVEF (left ventricular ejection fraction) and congestive heart failure, acute coronary syndrome and myocardial infarction, arterial thrombosis, and hypertension. Because of the need for long-term use of these agents, understanding the mechanism of cardiotoxicity and knowing which have cardiac toxicity are important [6].

- Imatinib targets Bcr-Abl, c-Kit, and PDGFR. Known side effects of Imatinib are peripheral edema, shortness of breath, and fatigue. Cardiotoxicity of Imatinib is controversial; several studies have observed no statistical differences between those treated with or without Imatinib; however, peripheral edema was more frequent in the Imatinib arm [7]
- Dasatinib targets Bcr-Abl, c-Kit, PDGFR, and Src family of kinases. Evidence of cardiotoxicity was seen early in clinical trials; in particular, pleural effusion and peripheral edema were described. The DASISION trial, which included 258 patients, one arm treated with Imatinib and the second with Dasatinib, has demonstrated significantly higher rate of pleural effusion and pulmonary hypertension in the Dasatinib arm [7]
- Nilotinib is an inhibitor of Bcr-Abl, c-Kit, and PDGFR. It can cause QT prolongation, leading sometimes to torsade de pointes. Ischemic heart disease is another complication shown in a clinical trial. After an average time on nilotinib therapy of 60 months, the incidences of ischemic heart disease-related cardiac events in the nilotinib 300 mg arm and 400 mg arm were 9.3 and 15.2%, respectively [7, 8]
- Ponatinib has the highest risk of cardiotoxicity from the TKIs, including congestive heart failure, cardiac arrhythmias, and hypertension. In the phase 2 ponatinib CML evaluation trial, ponatinib was shown to have dose-dependent cardiotoxicity in 267 evaluated patients. Among the ponatinib-treated CML patients participating in clinical trials, 31% reported arterial occlusive events in the 5-year follow-up. Additionally, 4% of patients reported cardiac adverse events of atrial fibrillation (AF) and 3% angina pectoris [8]

### 3.4. Cardiotoxicity of Immunomodulatory Drugs (IMIDs)

IMIDs are part of many multiple myeloma regimens, often in combination with other potentially cardiotoxic drugs, like proteasome inhibitors (PI). Both arterial and venous thrombotic events are described in association with IMIDs. The mechanisms of these side effects are direct damage of the endothelial cells, increased platelet aggregation, and higher von Willebrand factor levels [9]. Data from two phase III trials comparing combination of lenalidomide and dexamethasone to dexamethasone alone demonstrated an increased incidence of myocardial infarction and cerebrovascular events (1.98% and 3.4% vs. 0.57% and 1.7%, respectively) in the lenalidomide arm. Therefore, all patients should receive thromboprophylaxis with aspirin or, in case of high risk, anticoagulants [10]. IMIDs may also induce arrhythmias, like bradycardia or atrioventricular block, with thalidomide being associated to sinus bradycardia in 5% of patients [9, 11].

### 3.5. Cardiotoxicity of Proteasome Inhibitors

Proteasome inhibitors (PI) represent the backbone of multiple myeloma therapy. Inhibition of proteasomes induces apoptosis of the cells due to the aberrant proteome.

- Bortezomib is a first-generation PI. A systematic review and meta-analysis of cardiovascular adverse events (CVAE) in patients treated with Bortezomib showed a 3.8% rate of all-grade CVAE. However, randomised studies did not find a significantly higher risk of CVAE in the Bortezomib arm compared to the control arm [9]
- Carfilzomib is an irreversible proteasome inhibitor approved in 2012, and since then, there are increasing reports of carfilzomib-associated CVAE, including heart failure, hypertension, arrhythmias, ischemic events, and cardiac arrest. Possible mechanisms for these side effects are oxidative stress on myocardiocytes, endothelial effects, and an increased coronary vascular tone and reactivity. A meta-analysis of 24 prospective studies, including 2594 patients with multiple myeloma, showed a rate of all-grade CVAE of 18.1% and high-grade CVAE of 8.2%. Heart failure (4.15%) and hypertension (12.2%) were the most common side effects, while arrhythmias and ischemic events were less common. Higher doses of carfilzomib were associated with higher rates of CVAE [9]
- Ixazomib is an oral analog of Bortezomib, reversibly inhibiting the proteasome and the NFKB pathways in myeloma-supporting cells, influencing cytokines important for cell growth. Kumar et al. [12] reported an incidence of hypertension of 5% in patients treated upfront with combination of ixazomib, lenalidomide, and dexamethasone, but the TOURMALINE MM1 study, investigating the safety and efficacy profile of ixazomib, did not find significant differences in the incidence of CVAE between the ixazomib and placebo arms [13]

### 3.6. Cardiotoxicity of Immune Checkpoint Inhibitors (ICI)

Immune checkpoints have the role to prevent exaggerated immune response, while inhibition of them enhances immune activity, facilitating the antitumor immune response. They represent promising therapies in many refractory hematologic malignancies. Besides immune-related side effects, there are also cardiovascular adverse events described, like myocarditis, takotsubo syndrome, acute coronary syndrome, and pericardial disease [14].

## 4. Definition of Cardiac Dysfunction Secondary to Chemotherapy

Cancer therapy-related cardiac dysfunction is defined as a reduction of LVEF > 10% from baseline, with a LVEF lower than the normal limit. The cutoff for normality is considered 50%, but in patients treated with anthracyclines or trastuzumab, a LVEF in the low-normal range (50-55%) is associated with an increased risk of cardiotoxicity. Thus, the recommendation of the American Society of Echocardiography and the European Association of Cardiovascular Imaging is to consider 53% as the lower normal limit [1, 15, 16].

## 5. Evaluation of Cardiotoxicity Risk and Strategies of Prevention

In a large retrospective study including 820 cancer patients, 3.5% developed cardiac toxicity during the 10-year period, but there was no correlation between cardiac toxicity and traditional cardiovascular risk factors like age, sex, hypertension, diabetes, hyperlipidemia, obesity, and smoking. This raises the possibility of genetic predisposition for the development of cardiovascular toxicity [17].

Although there are no known predictive risk factors for the development of cardiotoxicity, a baseline risk assessment is mandatory in all patients before initiation of therapy, focusing on early, preclinical detection of cardiotoxicity. This would help to identify patients who could benefit from cardioprotective drugs and to adjust therapy before irreversible cardiac injury develops. Tests used to assess cardiac toxicity are cardiac imaging and biomarkers.

### 5.1. Cardiac Imaging for Early Detection of Cardiotoxicity

The goal of cardiac imaging is to assess cardiac structure and function and to identify early cardiac injury. This includes echocardiography, nuclear imaging, and magnetic resonance imaging (MRI).

Measurement of LVEF is a relatively insensitive tool for detection of early cardiotoxicity because important changes in LVEF occur only after a significant amount of myocardial damage is done and the compensatory mechanisms are overcome, but echocardiography is still widely used due to its availability and lack of radiation exposure. LVEF is routinely measured by echocardiography of multigated acquisition (MUGA). Although standard 2-dimensional (2D) echocardiographic assessment of LVEF has a higher interobserver and intraobserver variability than MUGA (8.8% vs. 6.8%), it offers additional information on valvular and diastolic function [18].

A disadvantage of 2D echocardiography is that the LVEF measurements depend on the quality of the images. The endocardial border has to be sufficiently visualised to track the end-systolic and end-diastolic volumes. The use of contrast agents can improve endocardial visualisation and reduce interobserver and intraobserver variability. Although several trials demonstrated the usefulness of contrast agents in the clinical practice, there are no clear indications of their use in the guidelines of the American Society of Echocardiography and the European Association of Echocardiography. Besides poor endocardial definition, other limitations of 2D echocardiography are ventricular foreshortening and the use of mathematical models and geometrical assumptions for calculating the LV volumes. Three-dimensional (3D) echocardiography can overcome these limitations, allowing a more accurate measurement of LV volumes and ejection fraction. Other advantages of 3D echocardiography are reduced analysis time, higher reproducibility, and lower interobserver variability. LV volumes obtained by 3D echocardiography correlate more closely with those obtained by computed tomography and MRI [18].

Another more sensitive tool for detection of early cardiac dysfunction is diastolic parameters. A study on 20 breast cancer patients with normal systolic functions has demonstrated that 50% of the patients treated with anthracyclines had impaired early peak flow velocity to atrial flow velocity ratio, deceleration time, and isovolumetric relaxation time [19]. A prospective study on 26 patients treated with anthracycline demonstrated an association between early alterations of diastolic parameters and the development of left ventricular dysfunction. Despite these observations, larger studies are needed to confirm the role of diastolic measurements in detection of cardiotoxicity.

Exercise and pharmacologic stress testing could also detect early changes in the LV function. A study on 37 patients treated with anthracycline revealed that an abnormal LVEF at rest after 1 month had a sensitivity of 53% and a specificity of 75% for detecting the risk of developing cardiac failure [19]. The addition of exercise increased the sensitivity to 89% but decreased the specificity to 41%. Another study on 23 patients with acute lymphoblastic leukemia treated with anthracyclines demonstrated a normal EF at rest but a reduced LVEF during stress [19]. Also, a study made on 49 patients with breast cancer revealed a subtle alteration of myocardial contractile function in 17% of them during low-dose dobutamine [20].

Myocardial deformation (strain) and deformation rate (strain rate) have the advantage over LVEF measurement to offer a multidimensional evaluation of myocardial mechanics and to detect subtle wall motion abnormalities that do not decrease LVEF. Several studies have demonstrated that strain and strain rate are more sensitive measures than LVEF for early detection of LV dysfunction [21–23]. A study on women treated with trastuzumab for breast cancer revealed that 51% of the patients had reductions in 2D longitudinal strain values and 37% reduction in 2D radial strain. Another study on 16 breast cancer patients treated with liposomal doxorubicin showed no changes in LV dimensions, LVEF, and systolic myocardial velocity at the end of chemotherapy, while longitudinal and radial strain and strain rates were significantly changed [24]. Strain measurements can also identify long-term effects of chemotherapy. In a cohort of 56 late survivors of childhood cancer treated with anthracyclines, strain measurements detected subclinical cardiotoxicity; both radial and longitudinal myocardial strain measurements were reduced by 15%, while LVEF remained normal [22].

Isotopic ventriculography is not currently used for monitoring cardiotoxicity due to the risk of ionizing radiation.

Cardiac MRI (CMR) can assess cardiac structure and function, and it can also evaluate pericardium, characterize myocardial tissue, and assess for cardiac infiltrates. CMR is a noninvasive method that offers a comprehensive assessment of myocardial function and myocardial tissue characterization, including assessment of strain, edema, and fibrosis. CMR can be used for LV chamber size quantification and systolic function measurement, providing quantification of chamber size and LVEF which is free from geometric assumptions and independent of acoustic windows. CMR myocardial tagging is also a well-established technique for measuring myocardial strain and was first described by Zerhouni et al. in 1988 [25]. Drafts et al. studied CMR parameters on cancer patients receiving anthracyclines before and 1, 3, and 6 months after therapy. After 6 months, LVEF decreased from 58 ± 1% to 53 ± 1% (*p* = 0.0002) and midwall circumferential strain from −17.7 ± 0.4 to −15.1 ± 0.4 (*p* = 0.0003) without evidence of focal fibrosis as defined by late gadolinium enhancement (LGE) [26]. CMR imaging with LGE is the reference standard for the noninvasive detection of focal myocardial fibrosis. Another advantage of CMR for evaluation of potential cardiotoxicity is the use of noncontrast parametric mapping techniques such as native T1 and T2 mapping, which rely on the intrinsic magnetic relaxation properties of the myocardium [27]. Immune checkpoint inhibitors often cause myocarditis, sometimes with fulminant evolution, which can also be diagnosed by CMR. In conclusion, CMR is a useful supplemental modality to echocardiography when a more reliable EF measurement is needed as well as for better tissue characterization [28].

### 5.2. The Role of Biomarkers in Early Detection of Cardiotoxicity

The poor sensitivity and variable reproducibility of LVEF measurements for detecting early cardiomyopathy have led to development of cardiac biomarkers. They offer an alternative solution for the shortcomings of imaging. There is no radiation exposure, and they are easier to perform than imaging. Several cardiac biomarkers have been proposed, the most studied ones being troponin and natriuretic peptides, reflecting cardiomyocyte damage and elevation in left ventricular filling pressure and wall stress, respectively. Other biomarkers are markers of inflammation: C-reactive protein (CRP), interleukin-6 (IL-6), and myeloperoxidase; of endothelial dysfunction: plasminogen activator inhibitor (PAI), tissue-type plasminogen activator (t-PA), and soluble intercellular adhesion molecule; and of myocardial ischemia: fatty acid binding protein, glycogen phosphorylase BB, and neuregulin-1 [29].

- Troponins: cardiac troponins (cTn) are markers of myocardial damage and they are released in response to ischemia, inflammation, oxidative stress, or apoptosis. They are the best studied markers of anthracycline cardiotoxicity. Increased cTn1 is present in one-third of patients treated with anthracyclines, and the proportion of patients with elevated cTn1 increases with the cumulative dose of anthracyclines. Elevation of cTn1 occurs early, within 12 hours in 53% of the patients. Therefore, measurement of cTn1 in the first 24 hours after treatment can detect early cardiotoxicity. cTn1 elevation can also predict late cardiac toxicity [30]. The pattern of cTn1 elevation offers prognostic information; in a study of 703 patients, a persistent cTn1 elevation 1 month after stopping anthracycline therapy was associated with higher incidence of cardiac events than in those with transient elevation [31]. Even before chemotherapy, in particular, patients with hematologic malignancies can have increased levels of cTn1, suggesting that the tumor itself can cause cardiac damage. cTn1 appears to have a higher predictive value than cTnT (troponin T), especially in leukemic patients [32]. Although troponins are sensitive and specific markers of cardiac injury, they can be elevated in other conditions too, like hypertensive emergency, renal failure, rhabdomyolysis, sepsis, and poor vascular health, thus limiting their use in predicting cardiotoxicity [33]
- Natriuretic peptides (B-type natriuretic peptide-BNP and its amino-terminal fragment-NT-pro-BNP) are markers of elevated left ventricular filling pressure and wall stress. Most of the studies have found a correlation between NT-pro-BNP elevation and cardiac dysfunction [29]. There is also a correlation between NT-pro-BNP and the cumulative anthracycline dose [34]. Patients with elevated NT-pro-BNP levels before chemotherapy had a higher risk of cardiotoxicity [35]. Similar to cTn1, elevation of BNP shortly after chemotherapy is a predictor for late cardiotoxicity. The pattern of elevation of BNP is also a prognostic factor; in a cohort of 52 patients treated with chemotherapy, persistently elevated NT-pro-BNP was strongly associated with development of cardiac dysfunction, compared to those with transient elevation, in whom no significant LVEF changes appeared during the 12-month follow-up [36]. A prospective study on 333 anthracycline-treated patients analyzed the predictive value of elevated BNP and LVEF obtained by MUGA for hospitalisation for congestive heart failure and mortality. This study found that both BNP and LVEF are independently predictive for congestive heart failure, but only BNP was associated with increased mortality. Future prospective trials are needed to standardize the use of BNP to diagnose patients with cardiac damage and to determine the optimal cutoff level and the timing for obtaining BNP samples. Also, future studies should focus on therapeutic decision-making according to BNP concentrations [37]. The use of natriuretic peptides for assessing cardiotoxicity has some limitations, evidence suggesting higher levels in the elderly and females, in case of renal failure, and the malignancy itself can increase BNP levels
- Markers of inflammation: studies have not demonstrated a direct correlation between inflammation markers like CRP, IL-6, and myeloperoxidase, but it can be assumed that changes in the antioxidant defence capacity may be associated with anthracycline-induced cardiotoxicity [29]. High-sensitivity CRP (hs-CRP) has been assessed for predicting cardiotoxicity in a study which included 49 women treated with trastuzumab. This trial showed a correlation between hs-CRP levels and the later onset of cardiomyopathy. Interestingly, hs-CRP levels appear to be higher in childhood cancer survivors, even if they were not exposed to cardiotoxic therapy, suggesting that hs-CRP is a marker of overall inflammation or tumor burden, in addition to chemotherapy effect [38]. Myeloperoxidase (MPO) is an enzyme produced by neutrophils and can lead to production of free radicals and to lipid peroxidation. One study showed that MPO levels after anthracycline administration correlated with the development of cardiotoxicity [39]
- Markers of endothelial dysfunction: activation of endothelium can lead to vascular dysfunction and accelerated atherosclerosis. A study on 90 patients with testicular cancer demonstrated higher levels of fibrinogen, CRP, von Willebrand factor, PAI-1, and t-PA in patients treated with chemotherapy, compared to those treated only with surgery. Those with higher PAI-1 levels had higher triglyceride levels, body mass index, and blood pressure and decreased carotid artery distensibility compared to controls. Increased levels of endothelial dysfunction markers suggest an increased risk of accelerated atherosclerosis [29, 30]
- Markers of myocardial ischemia: studies have demonstrated increased levels of fatty acid-binding protein (FABP) and glycogen phosphorylase-binding protein (GPBB) after chemotherapy, suggesting they could be a potential marker of cardiotoxicity [29]. In a study of patients treated with high-dose chemotherapy followed by stem cell transplantation, a group of patients with positive signal for GPBB was identified, without elevations of cTn or BNP; however it is difficult to demonstrate that GPBB is a more sensitive predictor for myocardial damage in the absence of long follow-up. Future larger trials are needed to assess the potential utility of GPBB [40]
- Neuregulin-1 (NRG-1) is a growth factor released by endothelial cells that bind to receptors on myocytes and stimulates cell growth, survival, and repair. A prospective study on 78 women treated with anthracycline for breast cancer showed a significant decrease of NRG-1 levels, suggesting the loss of this cardioprotective growth factor [29]
- Circulating microRNAs are short noncoding RNAs that play an important role in maintaining homeostasis, being implicated in regulation of oxidative stress response and cellular injury. Preclinical studies demonstrated increased levels of microRNAs (miR-146a) after doxorubicin administration [41]. Some microRNAs have been linked to specific cardiovascular diseases. The most investigated cardiac microRNAs are miR-1, miR-133, miR-208, and miR-499. A study involving 33 children demonstrated elevated miR-29b and miR-499 after anthracycline therapy, and the degree of elevation correlated with the anthracycline dose and troponin rise [42]. Another study involving breast cancer patients treated with doxorubicin revealed an increase in miR-1 which was strongly associated with LVEF reduction and was superior to troponin level in predicting cardiotoxicity [43]. MicroRNA level could be a marker specific for inflammatory or injury-mediated cardiotoxicity and heart failure; however, future studies are necessary for assessing the role of mIR-146a in chemotherapy-induced cardiotoxicity [41]
- Other novel emerging biomarkers are ST2, galactin-3, and growth differentiation factor 15 (GDF-15). There are only few studies investigating the potential role of these novel biomarkers in detecting chemotherapy-induced cardiotoxicity; some of them showed no significant association with cardiotoxicity; however, GDF-15 is an indicator of inflammation and oxidative stress and a promising parameter for detecting late cardiotoxicity. Future larger studies are needed to assess the role of these novel biomarkers [44]

## 6. Strategies to Prevent Cardiotoxicity

In order to reduce cardiotoxicity risk in cancer patients, several measures should be taken, including encouraging of a healthy lifestyle (regular exercise, healthy diet, and cessation of smoking) and identification and treatment of cardiovascular risk factors like dyslipidemia, increased glycated hemoglobin, and hypertension.

Other strategies to reduce cardiotoxicity include limiting the cumulative dose of cardiotoxic drugs and using less cardiotoxic regimens (liposomal anthracyclines).

The use of cardioprotective drugs is also a method to prevent/reduce cardiotoxicity. Cardioprotective agents used for prevention are as follows:

- Dexrazoxane
- Beta-blockers (carvedilol, nebivolol) that prevent LVEF reduction and decrease the incidence of heart failure
- Angiotensin-converting enzyme inhibitors (enalapril) that prevent LVEF deterioration during anthracycline therapy
- Combination therapies: in a paper published in 2016, the European Society of Cardiology recommended the use of cardioprotective drugs, like angiotensin-converting enzyme inhibitors/angiotensin II receptor blockers in association with beta-blockers. The OVERCOME trial demonstrated that patients who received enalapril and carvedilol had no reduction in LVEF at 6 months, compared to those who did not receive these drugs
- Statins that reduce cellular damage and heart failure risk during anthracycline treatment [1–3]

## 7. Monitoring Treatment-Related Cardiotoxicity

Initial evaluation of patients includes medical history and physical examination, electrocardiography, structural and functional evaluation (by echocardiography and biomarkers), risk stratification, and treatment of cardiovascular risk factors.

Monitoring during treatment should include transthoracic echocardiography at baseline and at the end of therapy (in case of TKIs, also every 3 months) and biomarkers (troponin +/- pro-BNP) before each cycle of therapy. Patients who present decreased LVEF or increased biomarkers at baseline or during therapy need cardiologic consultation and more frequent monitoring and, in selected cases, even adjustment of treatment [1].

## 8. Management of Therapy-Related Cardiotoxicity

- Heart failure (HF): asymptomatic patients with reduced LVEF need beta-blocker and ACE inhibitors to prevent clinical HF. They can be identified by elevated troponins or a decrease of global longitudinal strain > 15%. Chemotherapy withdrawal decisions should be made weighing the HF risk against the risk of cancer progression or relapse
- Hypertension is a common comorbidity in cancer patients and can be also caused by treatment, especially by VEGF (vascular endothelial growth factor) inhibitors. Monitoring blood pressure during therapy is important in order to prevent other complications, the target blood pressure being <140/90 mmHg in those with uncomplicated hypertension and <140/85 mmHg in those with diabetes or renal failure. The drugs of choice are ACE inhibitors, angiotensin receptor blockers, and beta-blockers. In case of poor control, amlodipine or aldosterone inhibitors could be added. Negative inotropes should be avoided due to the risk of HF
- Arrhythmias: both tachyarrhythmias and bradyarrhythmias can occur in chemotherapy patients and treatment includes rate control, sometimes anticoagulants and pacemaker implantation in case of symptomatic bradycardias
- Ischemic heart disease (IHD): patients treated with drugs associated with high risk of IHD (etoposide, bleomycin, vinblastine, etc.) should be closely monitored, and nitroglycerine or calcium antagonist should be given in case of angina
- Myocarditis and pericarditis are rare complications of chemotherapy, and their treatment follows the general recommendations
- Venous thromboembolic disease (VTD) is a common complication in cancer patients, caused by the malignancy itself but also favored by some treatments, like IMIDs, TKIs, and, in many cases, prophylactic treatment is necessary
- Pulmonary hypertension is seen mostly in patients treated with Dasatinib or Cyclophosphamide; therefore, these patients should be closely monitored with echocardiography
- Peripheral vascular disease: administration of nilotinib and ponatinib can be associated with arterial thromboembolism and early atherosclerosis, so correction of cardiovascular risk factors is important [1]

## 9. Long-Term Monitoring of Chemotherapy-Related Cardiotoxicity

Long-term follow-up is indicated for those patients who received a cumulative anthracycline dose of >250 mg/m^2^ or>35 Gy chest radiotherapy or a combination of anthracycline > 100 mg/m^2^ and radiotherapy > 15 Gy. Echocardiography is the method of choice for follow-up and should be performed 2 years after treatment and then every 5 years [1].

## 10. Guidelines for Management of Chemotherapy-Induced Cardiotoxicity

There are several guidelines regarding cardiotoxicity, proposed by the European and American cardiology societies. A cardio-oncology expert panel from the French Working Group of Cardio-Oncology analyzed the most recent American and European guidelines (American Society of Clinical Oncology (ASCO), European Society for Medical Oncology (ESMO), and European Society of Cardiology (ESC)) and proposed decision algorithms easy to use by clinicians in their daily practice.

All of the guidelines emphasize the need to identify patients with an increased risk of developing cardiovascular toxicity. Differences exist, but all of the definitions include patients with previous cardiovascular diseases, high-dose anthracycline, and combination therapy (Table 1).

The working group proposed the concept of the “cardio-oncological evaluation,” a global and standardized cardiovascular assessment strategy of patients with cancer, including risk factor assessment, ECG, biomarkers, and imaging evaluation (Table 2).

The working group also proposed an algorithm for management of cardiotoxicity.

- Management of overt treatment-related left ventricular systolic dysfunction (drop of LVEF with 10% to a value < 50% or a drop of 20%)

1. Asymptomatic patient: cardio-oncological evaluation and initiation of angiotensin-converting enzyme inhibitors (ACEI) and beta-blockers (BB)

- LVEF > 40%: in case of chemotherapy without anthracycline, continue the same treatment as long as the patient is asymptomatic + physical examination, transthoracic echocardiography, BNP, or NT-pro-BNP at 3 weeks then every 3 months. In case of anthracycline therapy, the same strategy as in those with LVEF < 40%
- LVEF < 40%: withhold therapy + physical examination, transthoracic echocardiography, BNP, or NT-pro-BNP at 3 weeks then every 3 months. In case of increasing LVEF, discuss resuming therapy

(2) Symptomatic patient (heart failure): cardio-oncological evaluation and initiation of angiotensin-converting enzyme inhibitors and beta-blockers, holding the involved cancer treatment and close cardio-oncological monitoring. In case of remission of symptoms (NYHA I), it can be discussed to restart therapy. In case of persistency of symptoms (NYHA II-IV), permanently stop the involved treatment

(B) Management of early cancer treatment-related myocardial toxicity: troponin rise > 99% of the upper reference limit and/or absolute global longitudinal strain (GLS) drop > 5% or ΔGLS > 12%

1. Troponin rise AND GLS drop > 5% or ΔGLS > 12%: cardio-oncological evaluation before the next administration and at 3 weeks initiate ACEI and/or BB. Cardio-oncological evaluation at 3 weeks and every 3 months unless symptoms develop. Continue the same treatment as long as no LVEF drop or symptoms
2. Troponin rise OR GLS drop > 5% or ΔGLS > 12%: cardio-oncological evaluation before the next administration and at 3 weeks discuss ACEI and/or BB. Cardio-oncological evaluation at 3 weeks and every 3 months unless symptoms develop. Continue the same treatment as long as no LVEF drop or symptoms [45]

## 11. Conclusions

Recent discoveries in oncology significantly improved overall survival of cancer patients, but they have also led to more complications of treatment. Some of these treatment-related complications are transient, but unfortunately, many have permanent impact on the quality of life and survival. Besides secondary malignancies, a life-threatening complication of cancer treatment is cardiac toxicity; therefore, a multidisciplinary approach is mandatory, to find a balance between the need for cancer cure and potential cardiotoxicity. As in other diseases, prevention is better than cure, hence the necessity to find methods with high sensitivity and sensibility to detect early, subclinical changes and allow prompt intervention to prevent further damages. Since imagistic methods are not able to detect early structural changes, cardiac biomarkers are promising parameters for early intervention. Although cardiac biomarkers, like troponin and NT-pro-BNP, have demonstrated their superiority over cardiac imaging, they are not routinely included in initial assessment and monitoring. A joint effort of oncologists and cardiologists is needed to elaborate guidelines for diagnosis and management of chemotherapy-related cardiotoxicity.

## Figures and Tables

**Table 1:** Patients with high risk of cardiotoxicity.

(i) High-dose anthracycline (e.g., doxorubicin ≥ 250 mg/m^2^ and epirubicin ≥ 600 mg/m^2^)
(ii) High-dose radiotherapy (≥30 Gy) if the heart is in the treatment field
(iii) Lower-dose anthracycline (e.g., doxorubicin < 250 mg/m^2^ and epirubicin < 600 mg/m^2^) or HER inhibitors or VEGF inhibitors or proteasome inhibitors of Bcr-Abl tyrosine kinase inhibitors and presence of any of the following factors:
(a) Age ≥ 60 years
(b) Lower-dose radiotherapy (<30 Gy) where the heart is in the radiation field
(c) ≥2 risk factors, including smoking, hypertension, diabetes mellitus, dyslipidemia, chronic renal insufficiency, and obesity
(iv) Previous heart disease
(v) Elevated cardiac biomarkers (pro-BNP, NT-pro-BNP, and troponin) before initiation of anticancer therapy

**Table 2:** Cardiovascular assessment included in the “cardio-oncological evaluation”.

(i) Clinical consultation (including blood pressure measurement)
(ii) ECG
(iii) Blood glucose, lipid profile, and glomerular filtration rate calculation
(iv) Cardiovascular global risk assessment using guidelines
(v) Transthoracic echocardiogram (TTE), including measurements of LVEF (ideally 3D but at least 2D) and GLS; in the absence of GLS quantification of LV longitudinal function, use mitral annular displacement by M-mode echocardiography and/or peak systolic velocity of the mitral annulus by pulsed-wave DTI
(vi) LV contrast agents could be potentially useful in 2D echocardiography
(vii) Cardiac magnetic resonance is recommended if the quality of TTE is suboptimal
(viii) Use the same imaging modality for monitoring
(ix) Actively manage modifiable cardiovascular risk factors and diseases
(x) Encourage exercise on a regular basis and healthy dietary habits

## Data Availability

The data supporting this systematic review are from previously reported studies and datasets, which have been cited. The processed data are available from the corresponding author upon request.
